# Exploring the value of a well-established conditioned pain modulation paradigm in women: a Translational Research in Pelvic Pain (TRiPP) study

**DOI:** 10.3389/fpain.2025.1439563

**Published:** 2025-03-12

**Authors:** Lysia Demetriou, Danielle Perro, Lydia Coxon, Michal Krassowski, Claire E. Lunde, Joana Ferreira-Gomes, Ana Charrua, Pedro Abreu-Mendes, Lars Arendt-Nielsen, Qasim Aziz, Judy Birch, Kurtis Garbutt, Andrew Horne, Anja Hoffman, Lone Hummelshoj, Jane Meijlink, Maik Obendorf, Esther Pogatzki-Zahn, Naoko Sasamoto, Kathryn Terry, Rolf-Detlef Treede, Allison Vitonis, Jan Vollert, Nilufer Rahmioglu, Christian M. Becker, Francisco Cruz, Stacey A. Missmer, Krina Zondervan, Christine B. Sieberg, Jens Nagel, Katy Vincent

**Affiliations:** ^1^ Nuffield Department of Women’s and Reproductive Health, Oxford Endometriosis Centre, University of Oxford, Oxforfd, United Kingdom; ^2^ Division of Adolescent & Young Adult Medicine, Department of Pediatrics, Boston Children’s Hospital, Boston, MA, United States; ^3^ IBMC/I3S and Faculty of Medicine of Porto Hospital S João, Porto, Portugal; ^4^ Department of Health Science and Technology, Center for Neuroplasticity and Pain (CNAP), SMI, School of Medicine, Aalborg University, Aalborg, Denmark; ^5^ Denmark and Department of Medical Gastroenterology, Mech-Sense, Aalborg University Hospital, Aalborg, Denmark; ^6^ Steno Diabetes Center North Denmark, Clinical Institute, Aalborg University Hospital, Aalborg, Denmark; ^7^ Centre for Neuroscience, Surgery and Trauma, Blizard Institute, Wingate Institute of Neurogastroenterology, Barts and The London School of Medicine and Dentistry, Queen Mary University of London, London, United Kingdom; ^8^ Pelvic Pain Support Network, Poole, United Kingdom; ^9^ MRC Centre for Reproductive Health, University of Edinburgh, Edinburgh, United Kingdom; ^10^ Research & Development, Pharmaceuticals Experimental Medicine, Bayer AG, Berlin, Germany; ^11^ Endometriosis.org, London, United Kingdom; ^12^ International Painful Bladder Foundation, Amsterdam, Netherlands; ^13^ Department of Anesthesiology, Intensive Care and Pain Medicine, University Hospital Muenster, Muenster, Germany; ^14^ Department of Obstetrics and Gynaecology, Brigham and Women’s Hospital and Harvard Medical School, Boston, MA, United States; ^15^ Boston Center for Endometriosis, Brigham and Women’s Hospital and Boston Children’s Hospital, Boston, MA, United States; ^16^ Department of Epidemiology, Harvard T.H. Chan School of Public Health, Boston, MA, United States; ^17^ Department of Neurophysiology, Mannheim Center for Translational Neuroscience (MCTN), Heidelberg University, Mannheim, Germany; ^18^ Department of Clinical and Biomedical Sciences, Faculty of Health and Life Sciences, University of Exeter, Exeter, United Kingdom; ^19^ Wellcome Centre for Human Genetics, University of Oxford, Oxford, United Kingdom; ^20^ Department of Obstetrics, Gynecology, and Reproductive Biology, College of Human Medicine, Michigan State University, Grand Rapids, MI, United States; ^21^ Department of Pediatrics, Division of Adolescent and Young Adult Medicine, Boston Children’s Hospital and Harvard Medical School, Boston, MA, United States; ^22^ Department of Psychiatry, Center for Health Outcomes & Interdisciplinary Research, Massachusetts General Hospital, Boston, MA, United States; ^23^ Department of Psychiatry, Harvard Medical School, Boston, MA, United States; ^24^ Exploratory Pathobiology, Research & Development, Pharmaceuticals, Bayer Aktiengesellschaft, Wuppertal, Germany; ^25^ Nonclincal Sciences & Operations, Merz Therapeutics, Frankfurt, Germany

**Keywords:** conditioned pain modulation, chronic pelvic pain, quantitative sensory testing, women's health, pain characteristics

## Abstract

**Background:**

Conditioned pain modulation (CPM) is considered a human proxy for descending inhibitory pain pathways. However, there is wide variation in the CPM response described in the literature and ongoing debate about its utility.

**Methods:**

Here we explored CPM in women with (*n* = 59) and without (*n* = 26) chronic pelvic pain (CPP), aiming to determine the magnitude of effect and factors influencing variability in the CPM response.

**Results:**

Using a pressure pain threshold test stimulus and ischaemic pressure cuff conditioning stimulus (CS), we found no significant difference in the mean CPM effect between CPP and control participants. Using a robust statistical method (+/−2 standard error of measurement) to further investigate CPM, there was no significant difference in the proportion exhibiting inhibition between controls and CPP participants (*X^2^* = 0.003, *p* = 0.96). Notably, only 23.1% of our healthy controls demonstrated a “true” CPM effect (*n* = 4 inhibitory, *n* = 2 facilitatory). Despite a rich data set, we were unable to identify any single questionnaire, clinical or psychophysical covariate correlating with the CPM effect.

**Conclusions:**

Despite using one of the recommended CPM paradigms we were only able to demonstrate “true” CPM in 23.1% of control participants. Thus, the absence of differences between women with and without chronic pelvic pain must be interpreted with caution. Future studies using different CPM paradigms or larger sample sizes may find different results. Although CPM in chronic pain populations is of major theoretical mechanistic interest, the lack of an established assessment standard led us to question its added value in current clinical research.

## 1 Introduction

Conditioned pain modulation (CPM) is considered a human proxy for the diffuse noxious inhibitory control mechanism found in rodents. It describes decreased pain after application of a painful conditioning stimulus in a distant site, supporting the idea that pain inhibits pain (1). The CPM paradigm is a psychophysical/neurophysiological test that is assumed to mimic the function of endogenous pain pathways responsible for the balance between pain inhibition and facilitation. Dysfunction in endogenous pain pathways has been proposed as one of the mechanisms underlying chronic pelvic pain (2) and other chronic pains (fibromyalgia and chronic widespreadness pain).

A wide variety of studies show differences between patients and controls. However, this is not true for all studies, in sex disaggregated chronic pain cohorts, reported CPM differences between patients and healthy controls vary. In females with irritable bowel syndrome (IBS), CPM impairment differed between disease severity subtypes, but not between those with and without IBS (3); similar findings were seen in association with primary dysmenorrhoea (4, 5). Contrarily, women with comorbid primary dysmenorrhoea and bladder pain sensitivity demonstrated decreased CPM efficiency compared to both other pain groups and controls (6).

Importantly even among healthy controls, variability in the CPM response occurs and different assessment paradigms produce different responses in the same individuals (7). While it is expected that relative to chronic pain participants, healthy controls will exhibit greater CPM inhibition, there is (conflicting) evidence for factors causing variation in the individual CPM effect, including age, sex and menstrual phase (8, 9). Our understanding of participant characteristics impacting CPM remains limited and inconclusive.

Growing evidence suggests that paradigm parameters such as stimuli modality and site of application contribute to variation in CPM effect (10, 11). Whilst well-established CPM paradigms exist, there is no consensus on which is the gold standard (12). Consequently, paradigm inconsistency poses a challenge when comparing CPM results across studies. Recent efforts to improve the standardisation and comparability of CPM testing and reporting have proposed that the reliability of the CPM effect is an important first step (13, 14). A robust statistical approach, considering the standard error of measurement (SEm, a combined measure of standard deviation and reliability) when interpreting CPM effect, is also recommended (15). Kennedy et al. suggest that any CPM response ≥2SEm or ≤−2Sem can be seen as a “true” change at the individual level, while responses between these thresholds should be interpreted as signal noise (14). Additionally, as per the Jacobson's criterion for reliable change of clinical significance beyond measurements error and random chance, it is recommended to report data as the distribution of responders and non-responders at an individual rather than a group mean level (16, 17), due to the risk of inhibitory/facilitatory responders and non-responders cancelling each other out (18, 19).

Here we evaluate the utility of CPM in women with chronic pelvic pain (CPP) and in healthy controls. We aimed to: (1) identify the frequencies of “true” CPM effect in each group, (2) assess intrasession reliability and (3) investigate the relationship between CPM and participant characteristics.

## 2 Methods

### 2.1 Study population

Participants for TRiPP, were identified from two existing endometriosis cohort studies in Oxford (EndOX: A study to identify possible biomarkers in women with endometriosis, Oxford REC ref 09/H0604/58) (*N* = 276) and Boston [The Women's Health Study from Adolescence to Adulthood (A2A), IRB-P00004267] (*N* = 494), while 16 BPS participants were recruited at Hospital São João/Instituto de Biologia Molecular e Celular (IBMC) in Porto (20). The present study was conducted with a subset of these participants across the three sites, after obtaining all necessary ethical approval (Ethics Reference: 19/YH/0030). All participants were compensated for their time and participation in accordance with the specific requirements and regulations for clinical studies at each site.

A subset of the cohort participated in psychophysical pain testing (*N* = 85), in addition to completing comprehensive questionnaires, as illustrated in the study flow chart (21) (Supplementary Figure S1, Phase III). Female participants invited for psychophysical testing either had an indication of chronic pelvic pain (CPP) for at least three months (chronic pelvic pain syndrome, endometriosis-associated pain, bladder pain syndrome, or comorbid bladder pain syndrome & endometriosis) with at least one pelvic pain rated > = 4/10 or were controls without pelvic pain (CON) (no/minimal pelvic pain including dysmenorrhoea, all NRS <3/10). In addition to the pelvic pain rating (>=4/10), inclusion criteria for the participants in the CPP group included a surgical confirmation of endometriosis and/or urinary symptoms and pelvic pain perceived as arising from the bladder. Participants in the control group needed to have no history of endometriosis and no urinary symptoms. Recruitment was restricted to females aged 18–50 who were neither pregnant nor lactating. CPP participants were combined into a single group, irrespective of underlying pathology, for the purposes of the analyses described in this manuscript. Increasing awareness in the field of the similarities between chronic pain conditions (22) that are also represented in the ICD-11 (Code:MG30), justify our heterogenous CPP group in terms of the underlying pathology and its clinical presentation, whilst our focus is on the pain symptoms.

All researchers underwent coordinated training, performed the paradigms according to a script and used identical equipment to ensure consistency of data across sites. Due to their extended interactions with the CPP participants, it was not possible for the researchers to remain blinded as to which group the participants belonged. This project was pre-registered on clinicaltrials.gov: NCT04001244.

### 2.2 Study design

Valid informed consent was obtained prior to commencing the physiological testing study visit. All participants completed comprehensive validated questionnaires and undertook a variety of psychophysical tests [quantitative sensory testing (QST)], CPM, recordings of autonomic nervous system (ANS) activity [via electrocardiogram (ECG) and blood pressure recordings] and non-invasive bladder testing) (20). Prior to any psychophysical testing, all participants completed a bespoke “How are you today?” questionnaire, comprising validated questionnaire measures assessing variables potentially influencing CPM: current pain intensity, and location (body map), anxiety [State-Trait Anxiety Inventory (STAI)-State questionnaire], pain catastrophising scale (PCS), menstrual cycle status, use of exogenous hormones, medication and caffeine use in the last 24 h (23, 24). The current bodily pain intensity was assessed with the question “How would you assess your pain now, at this moment?” and participants were asked to answer by using an 11 point NRS scale where 0 = “no pain at all” and 10 = “worst imaginable pain”. This was followed by the Michigan whole a body map on which participants were asked to mark the location(s) of their pain. Prior to the visit, participants were instructed to refrain from taking analgesic medications and reduce caffeine consumption for the previous 24 h. Time of CPM visit was recorded.

### 2.3 Questionnaire measures

#### 2.3.1 Validated questionnaires—pain catastrophizing scale and state-trait anxiety inventory

Both the Pain Catastrophizing Scale (PCS) (α=0.92) and the State section of the State-Trait Anxiety Inventory-State (STAI-S) (α=0.31−0.86) are self-administered questionnaires, with poor to excellent test-retest reliability, in part owing to the time between the test and retest (25, 26). The PCS is a 13-item questionnaire which assesses one's negative perception of their pain, and is characterised by three subscales; rumination, magnification and helplessness (27, 28). A higher PCS score indicates greater catastrophizing, and a score above 30 is of clinical significance. The STAI-S is a measure of state anxiety, or one's anxiety levels at the time. The questionnaire comprises 20-items, and a score above 40 indicates anxiety at a clinically significant level (23). Questionnaires were scored according to standardised protocols (25, 27).

#### 2.3.2 Pain location

The Michigan body map was used to determine the number of extra-pelvic regions impacted by pain. Participants were categorised according to previously published methodology from MAPP (27) into: isolated (0 additional regions), intermediate (1–2 additional regions), widespread (3–7 additional regions).

### 2.4 Conditioned pain modulation paradigm

Participants were tested in a temperature-controlled room, maintained at 20°C. To assess CPM, a force dial 10 kg algometer with a 1 cm^2^ rubber tip test stimulus (TS) was applied three times to the right dorsal foot increasing at a rate of 0.5 kg/cm^2^ per second. The applied pressure (measured in kg) was recorded after each application (29). The mean of the three pressures was calculated to determine the baseline pressure pain threshold (PPT1_average_), as described by the German Research Network on Neuropathic Pain (DFNS) (29). A pressure cuff conditioning stimulus (CS) was applied to the left arm; the cuff was pumped up at a rate of ∼20 mmHg/second until participants identified the stimulus as painful. The cuff was maintained at that pressure for 60 s. Prior to deflation, the participant was asked to rate the pain elicited from the CS out of 10 (0 = not painful, 10 = worst pain imaginable). The self-reported pain rating and CS pressure were recorded. Immediately after deflation, the algometer was again applied to the right dorsal foot three times, and the mean PPT2_average_ was calculated.

Participants were then instructed to rest quietly for 10 min, prior to repeating the application of the CS and TS. Pressure, pain rating of the CS prior to deflation, and the mean PPT3_average_ TS were again calculated.

### 2.5 Data preparation and analysis

#### 2.5.1 Determining CPM effect

In line with recommended reporting, CPM effect was calculated and reported as both the absolute difference in pressure pain threshold (PPT2_average_-PPT1_average_) and percentage change (PPT2_average_-PPT1_average/_PPT1_average_)x100 (13). When determining the “true” CPM effect, the absolute difference was used.

#### 2.5.2 Standard error of measurement and CPM reliability

Using CON PPT1 recordings, standard error of measurement (SEm) was calculated using the formula below. Results from an analysis of variance (ANOVA) using PPT1 recordings were used to determine reliability in the SEm equation.

$$\mathrm{Reliability}\;\mathrm{baseline}\;\mathrm{PPT}=\frac{\mathrm{Residual}\;\mathrm{sum}\;\mathrm{of}\;\mathrm{squares}}{\mathrm{degrees}\;\mathrm{of}\;\mathrm{freedom}}$$

SEM=standarddeviationofbaselinePPT×√(1−reliabilitybaselinePPT)

As described, we used a threshold of +/−2SEm to determine a “true” CPM effect (14, 30). The absolute difference in PPT2_average_—PPT1_average_ was compared against the +/−2SEm threshold. A score >+2SEm was considered an inhibitory CPM response, and a score <−2SEm was considered a facilitatory response. Scores between the thresholds were classified as “non-responders”.

Intrasession reliability of the effect in healthy controls and CPP participants was determined separately using the kappa statistic (k, SE) (31, 32). Participants were classed as responders (either facilitatory or inhibitory) or non-responders, as described above using data from PPT2_average_ and PPT1_average_. The CPM effect was calculated again using the PPT3_average_—PPT1_average_. Participants were similarly classed by response types. A 3 × 3 table was used to generate the kappa statistic. Reliability was additionally calculated using CPM effect as a continuous variable, using the residual sum of squares equation above to assess reliability between PPT3_average_—PPT1_average_ and PPT2_average_ -PPT1_average_. This was calculated as follows:

$$\mathrm{Reliability}=\frac{\mathrm{Residual}\;\mathrm{sum}\;\mathrm{of}\;\mathrm{squares}}{\mathrm{Degrees}\;\mathrm{of}\;\mathrm{freedom}}$$

#### 2.5.3 Factors that may influence the CPM response

##### 2.5.3.1 Menstrual phasing

On the day of the CPM visit, participants were asked to self-report whether they were taking any hormonal contraceptives, the day of their last menstrual period (LMP), and typical length of their menstrual cycle. Those who were not currently using hormonal contraception, who indicated that they still had menstrual cycles, were categorised by menstrual phase according to the following protocol (32). Based on a 28-day cycle, day 1–7 were classified as menstrual phase, day 8–14 were classified as follicular/proliferative and day 15 + as luteal/secretory phase. For participants whose cycle length deviated from 28 days, 14 days were subtracted from their reported cycle length, the secretory phase being held constant, and the remaining duration was assigned to proliferative phase. For females who reported a cycle length range (i.e., 40–45 days), the cycle length was calculated using the minimum, mean or maximum cycle length reported. From these three measures, participant phase was allocated based on which calculated phase was most common. Menstrual phase was cross-checked by two researchers to ensure consistency.

##### 2.5.3.2 Pain rating and pressure (mmHg) of the pressure cuff

The self-reported pain intensity rating (0–10 NRS/Visual Analogue Scale) and pressure (mmHg) of the CS was compared between CPM response groups.

##### 2.5.3.3 Time of participant visit and medication use

The start and end time of the CPM visit were recorded by the researcher. The scripted paradigm took approximately one hour. Self-reported medication use (primarily centrally-acting medications, analgesics and antihistamines) was summarised for CPP and control participants separately, and the number and percentage of each group not currently taking medications were tabulated and compared using a Fisher's exact test.

##### 2.5.3.4 Quantitative sensory testing

The full DFNS Quantitative Sensory Testing (QST) protocol was performed on the dorsum of the right foot (control site) (29) as well as on the lower abdomen/pelvis (test site) which is not used here (32) The full QST paradigm includes both non-nociceptive measures (such as cold detection threshold, warm detection threshold, thermal sensory limen, mechanical detection threshold, vibration detection threshold) and nociceptive measures (cold pain threshold, heat pain threshold, mechanical pain threshold, mechanical pain sensitivity, dynamic mechanical allodynia, wind up ratio, pressure pain threshold) and full results of this in this study have been previously published (32). The QST script was translated into Portuguese for participants at IBMC. All QST sessions were carried out in an air-conditioned room at approximately 20°C. Before the session, participants were asked to complete the “How are you today?” questionnaire. All but eleven CPP participants completed the QST and CPM paradigms within a single visit (20).

### 2.6 Statistical analyses

Descriptive frequencies and distributions of the “How are you today?” questionnaire responses were calculated. Continuous variables were assessed for normality using the Shapiro–Wilks test. Comparisons between the pain and control groups were made using an unpaired *t*-test, or where appropriate, the Mann–Whitney test. Mean [standard deviation (SD)] or median [interquartile range (IQR)] were reported, depending on whether the data were normally distributed.

To further investigate the relationship between the CPM response and both phenotypic characteristics and characteristics of the paradigm itself, Spearman's correlations were performed. In the case where such measures did not differ significantly between control and CPP participants, participants were combined for the correlation analysis to increase power.

All QST data were collected using the official DFNS QST form and later uploaded to a secure database. Data inputting was independently verified by two researchers. Published reference data were used to Z transform the data for the foot (29). A z-score greater than 0 indicates a gain of function and a z-score less than 0 shows a loss of function. A z-score of above 1.96 or below −1.96 would be outside of the 95% confidence intervals of the normal distribution of the healthy reference data, which has a mean of zero and standard deviation of one. Mean QST z-scores for all nociceptive and all non-nociceptive QST parameters were calculated for each response group (inhibitory, facilitatory) separately in controls and CPP participants. T-tests were performed to compare between response groups, for controls and CPP participants separately. The Bonferroni *p*-value threshold for multiple comparisons was 0.05/4 = 0.0125. Data were analysed and plotted using Graphpad Prism 9.

All phenotypic data were analysed and plotted using Graphpad Prism 9. In all plots, the diamond shape identifies control participants, whereas the circle identifies CPP participants. Furthermore, blue shapes indicate that the participant exhibited CPM facilitation, whereas red shapes indicate that the participant exhibited CPM inhibition.

## 3 Results

### 3.1 Participant characteristics

CPM was performed on 85 women; *n* = 59 with CPP and *n* = 26 CON. All CON participants and twenty-four CPP participants were recruited from Boston, USA. Twenty-four CPP participants were recruited from Oxford, UK, and eleven participants from Porto, Portugal. See Table 1.

**Table 1:** Participant characteristics (CPP, chronic pelvic pain participants; IQR, interquartile range; CPM, conditioned pain modulation) and conditioned pain modulation parameters (CPP, chronic pelvic pain participants; PPT, pressure pain threshold; CS, conditioning stimulus; u, SD; mu/mean, standard deviation. Kg, kilograms; mmHg, millimetres of mercury).

Characteristic	CPP (*n* = 59)	Controls (*n* = 26)	*p*-value [confidence intervals (CI)]
Age (median, IQR)	34 (13)	29.5 (12.5)	0.124 [−1.0, 7.0]
BMI (median, IQR)	24.28 (7.86)	22.65 (3.73)	0.068
Participants from Each Site (*n*, %)			
Boston, USA	24 (40.7)	26 (100)	
Oxford, UK	24 (40.7)	0	
Porto, Portugal	11 (18.6)	0	
Number (%) of participants using a form of hormonal contraceptives	43 (72.9)	12 (46.2)	0.0262
Number (%) of participants currently having menstrual cycles	12 (20.3)	14 (53.8)	0.0042
Caffeinated beverage consumption on day of CPM visit [cups, median (IQR)]	0 (1)	0 (1)	0.161
Number (%) of participants that have smoked more than 100 cigarettes during their lifetime	6 (11.8)	3 (13.6)	0.549
PCS Score (median, IQR)	14.5 (18.5)	1.5 (8)	<0.0001
*N* (%) meeting clinical cut-off	10 (17.0)	0 (0)	–
STAI-S Score (median, IQR)	31 (10.5)	23 (23)	0.0032
*N* (%) meeting clinical cut-off	12 (20.3)	2 (7.7)	–
Parameter			
Pain rating before CPM paradigm (/10 NRS scale) (median/SD)	2 (5)	0 (1)	<0.0001 [−194.0, −167.0]
Change in PPT (kg)	0.47 (0.413)	0.5 (0.483)	0.795
[after CS (μ, SD)]			
% Change in PPT (%)	12.74 (15.08)	11.88 (11.12)	
Pressure cuff pain rating (Median, IQR)	5 (5)	3 (3)	0.0070 [0.500, 3.00]
Pressure (mmHg) of CS (μ, SD)	202.5 (63.49)	161.9 (40.10)	0.0036 [0, 2.0]

There was no significant difference in age, BMI or number of caffeinated beverages consumed between the controls and those with CPP. However, there was a significant difference in the current pain intensity between the CPP group and controls [median: 2(SD: 5) vs. median: 0 (SD: 1), *p* < 0.0001], and a smaller proportion of CPP participants were having a menstrual cycle (20.3% vs. 53.8%, *p* = 0.0042) as they were more likely to be using hormones to induce amenorrhoea therapeutically. Forty-three (72.9%) CPP participants and only *n* = 12 (46.2%) control participants were using hormonal contraceptives at the time of the CPM visit (*p* = 0.026). The median (IQR) PCS scores for CPP participants and controls were 14.5 (18.5) and 4.5 (8) respectively (*p* < 0.0001). Ten CPP participants exceeded the clinical cut-off for PCS (> = 30), whereas no control participants met this cut-off. The median (IQR) STAI-S scores for pain participants and controls were 31 (10.5) and 23 (23) respectively (*p* = 0.0032). Twelve CPP participants and two CON exceeded the clinical cut-off for STAI-S (> = 40).

### 3.2 CPM effect and testing parameters

The mean (SD) absolute difference in PPT_average_ (kg) before and after the CS was 0.47 (0.41) and 0.5 (0.48) for CPP participants and controls, respectively. The mean (SD) % change was 12.74 (15.08) and 11.88 (11.12) for CPP participants and controls, respectively (*p* = 0.795). As demonstrated in Table 1, there was a significant difference between the median (IQR) pain rating of the CS [CPP = 5(5), CON = 3 (3) *p* = 0.0070] and mean (SD) pressure (mmHg) of the CS between CPP participants and controls [CPP=202.5 (63.49), CON=161.9 (40.10), *p* = 0.0036].

### 3.3 Standard error of measurement (+/−2Sem) threshold and reliability

**Figure 1:**
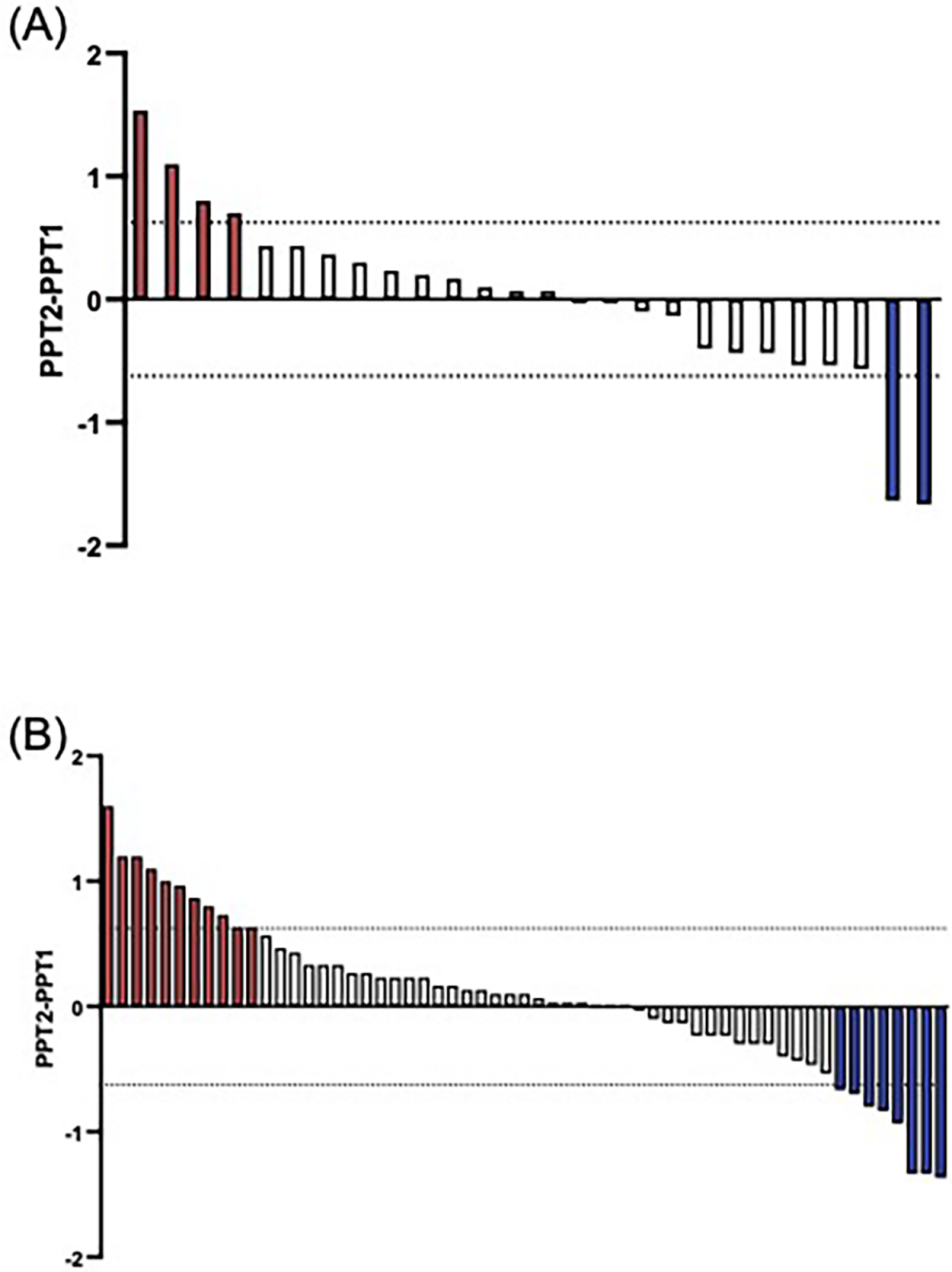
Participants with a “true” CPM effect using the +/−2SEM threshold. There is wide variation in the CPM response amongst healthy control participants. Red bars indicate an inhibitory response, and blue, a facilitatory response. Each bar represents a single participant. **(A)** True CPM effect for controls, *n* = 4 exhibited an inhibitory response, and 2 a facilitatory response. **(B)** True CPM effect for pain participants, *n* = 11 exhibited an inhibitory response, and 8 a facilitatory response.

The +/−2SEm threshold was determined to be +/−0.624. When comparing the absolute difference in PPT against this threshold, six (23.1%) controls (*n* = 4 inhibitory, *n* = 2 facilitatory, Figure 1A) and nineteen (32.2%) CPP participants (*n* = 11 inhibitory, *n* = 8 facilitatory, Figure 1B) had a “true” CPM effect. There was no significant difference in the proportion exhibiting inhibition between the two groups (*X^2^* = 0.003, *p* = 0.96). Using a categorical approach, intrasession reliability of the CPM response in controls [k, SE: 0.193 (0.145)] was poor, and fair in CPP participants [k, SE: 0.348 (0.111)]. Using CPM effect as a continuous variable, the intrasession reliability was fair for both controls (k = 0.295) and CPP participants (k = 0.353).

### 3.4 Investigating the relationship between participant characteristics and the CPM effects

#### 3.4.1 Validated questionnaire measures: PCS and STAI-S

**Figure 2:**
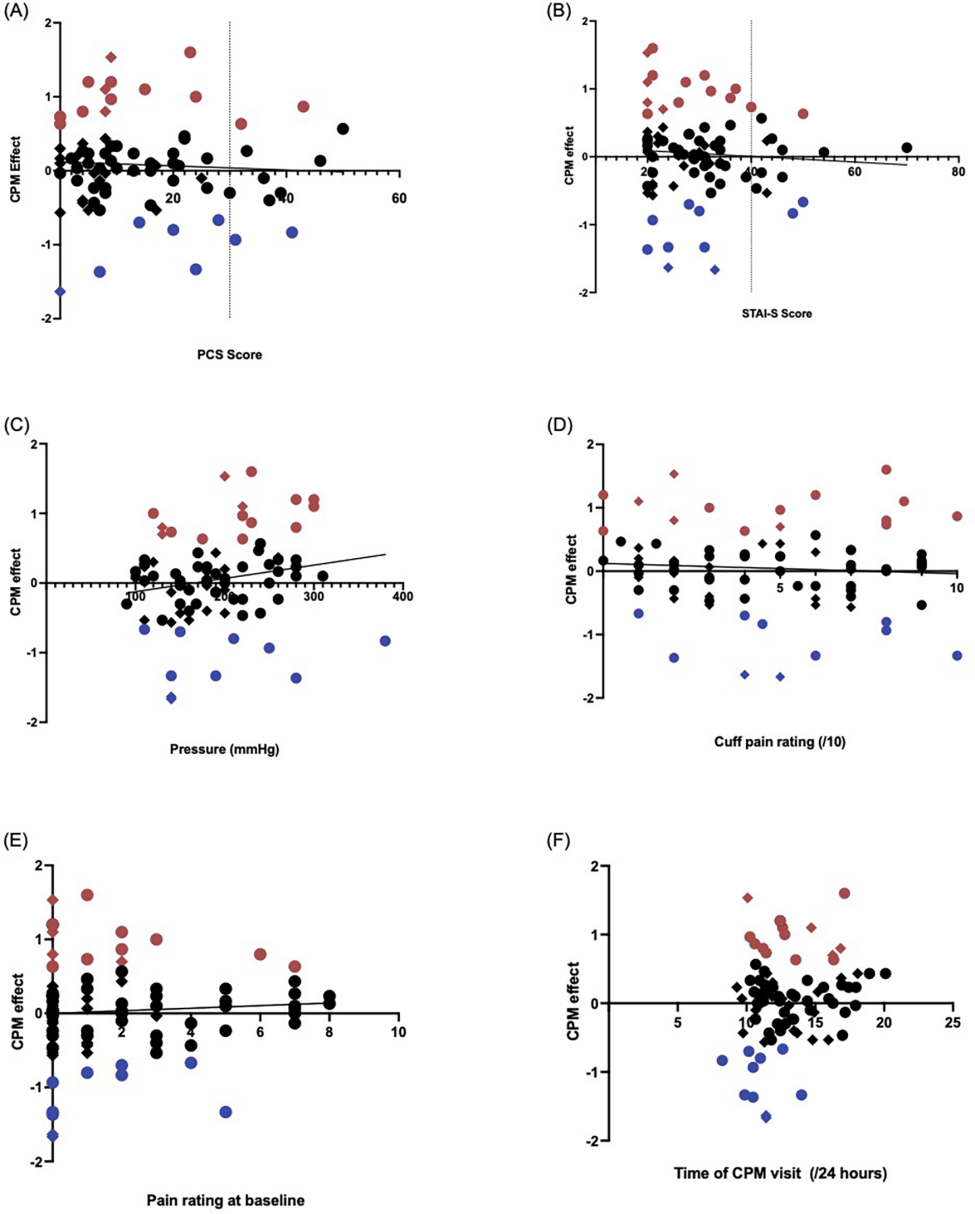
Validated questionnaire measures which assess mood showed no significant correlation with the CPM effect when data from all participants were included. In each figure, the blue dots represent participants with a facilitatory CPM response, and red dots are participants with an inhibitory CPM response. Circles identify CPP participants, and diamonds indicate healthy controls. **(A)** Pain catastrophizing scale (PCS) score by CPM effect (PPT2-PPT1). There was no correlation between the CPM effect and PCS score (*rho*=−0.0552, *p* = 0.631). All ten participants exceeding the clinical cut-off of x = 30 for PCS were CPP participants. **(B)** State-trait anxiety inventory state (STAI-S) score by CPM effect. There was similarly no correlation between the STAI-S measure and CPM effect (*rho*=−0.0881, *p* = 0.426). All but two participants whose STAI-S scores exceeded the clinical cut-off of y = 40 were in the CPP group. **(C,D)** Elements of the pressure cuff conditioning stimulus–correlation with the CPM effect in CPP and control participants. Red shapes indicate participants exhibiting CPM inhibition, and the blue shapes indicate those with CPM facilitation. Circles; CPP participants, Diamonds; Control participants. **(C)** Pressure (mmHg) at which participant identified that the stimulus was painful, before it was maintained at that pressure for 60 s (rho=0.207, *p* = 0.059). **(D)** Self-reported pain rating of the conditioning stimulus (/10), recorded after the 60 s maintenance of the pressure cuff, just prior to release of the pressure cuff (rho=−0.065, *p* = 0.557). **(E)** Self-reported pain at baseline before the CPM session began (/10) (rho=0.071, *p* = 0.525). CS, conditioning stimulus; SEM, standard error of measurement. **(F)** Correlation between the time of day (on 24-hour time scale) participants began the CPM visit and CPM response. Red shapes indicate participants with CPM inhibition, and the blue shapes indicate those with CPM facilitation. Circles; CPP participants, Diamonds; Control participants. CPM, conditioned pain modulation; PPT, pressure pain threshold.

No significant association were found between the CPM effect and the PCS scores (*r* = −0.056, *p* = 0.63) (Figure 2A) or STAI-S scales (*r* = −0.088, *p* = 0.43) (Figure 2B).

#### 3.4.2 Hormone use and menstrual phase at the time of CPM visit

Our sample size was not large enough to allow statistical exploration of the relationship between hormone use or menstrual cycle phase and CPM response, however no clear pattern was seen. Three (75%) of the inhibitory responders, twelve (60%) non-responders and one (50%) facilitatory responder were currently using hormonal contraceptives. Fourteen of all CON participants had a natural menstrual cycle (i.e., were not currently on any form of hormonal contraceptives) at the time of the CPM visit, two of which did not provide menstrual cycle data. Only one (25%) inhibitory responder, four (20%) non-responders and zero facilitatory responders were currently in the follicular phase of the menstrual cycle.

#### 3.4.3 Pain location

No significant associations were found between the CPM effect and widespread pain characterisation (*r* *=* *−0.030, p* *=* *0.82*).

#### 3.4.4 Pain ratings and pressure cuff measurement

**Figure 3:**
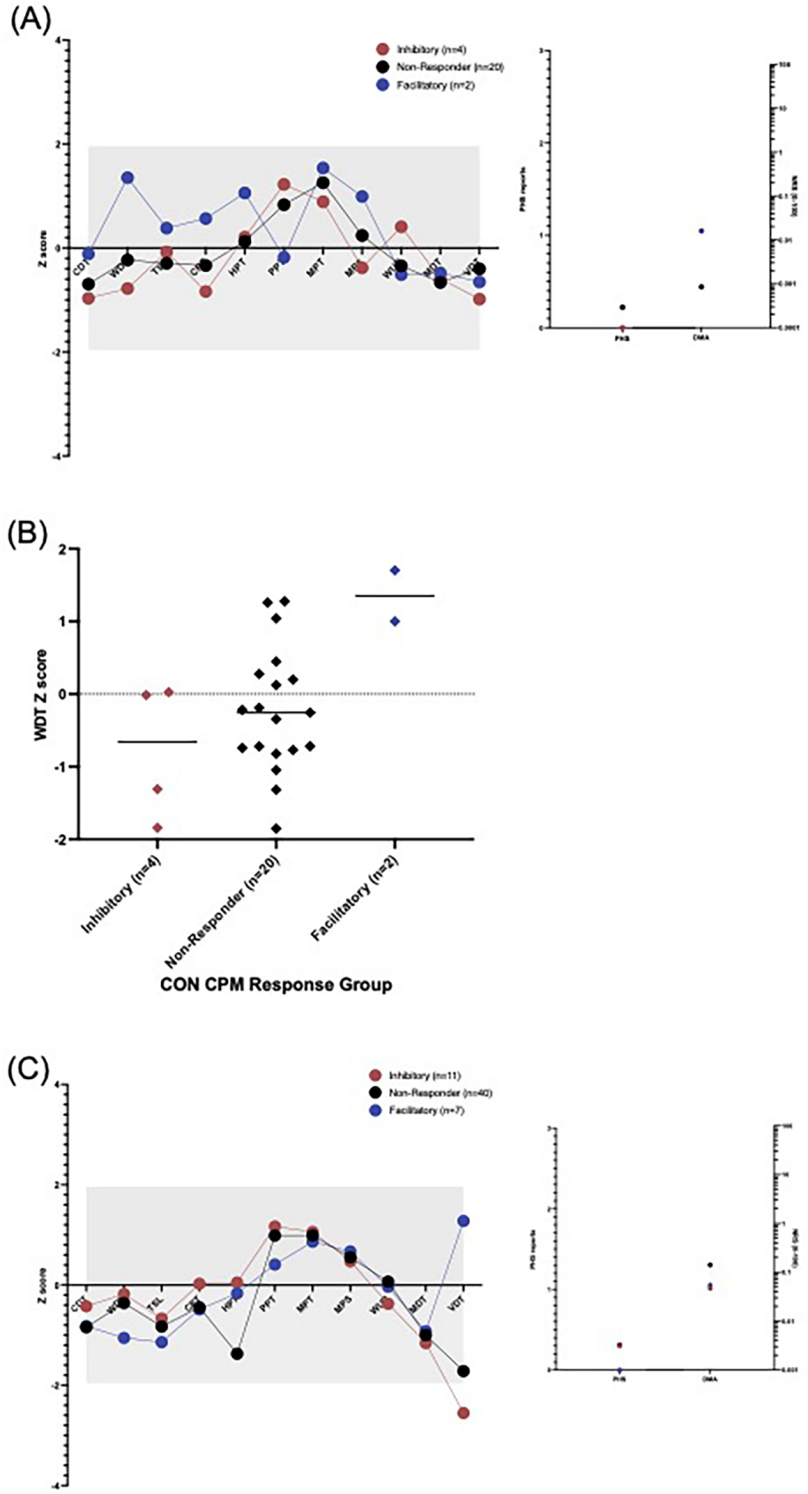
Quantitative sensory testing profile of **(A)** control participants and **(B)** CPP participants by CPM response group (red = inhibitory, black-non-responder, blue = facilitatory). A significant gain of function is indicated if the dot is above grey area (Z>+1.96), and a loss of function; a dot below the grey area (Z<−1.96). CDT, cold detection threshold; WDT, warm detection threshold; TSL, thermal sensory limen; CPT, cold pain threshold; HPT, heat pain threshold; PPT, pressure pain threshold; MPT, mechanical pain threshold; MPS, mechanical pain sensitivity; WUR, windup ratio; MDT, mechanical detection threshold; VDT, vibration detection threshold; PHS, paradoxical heat sensations (count) and DMA; dynamic mechanical allodynia (NRS 0-100) would not be expected to be seen in healthy participants. **(C)** WDT differences between control participants exhibiting CPM inhibition and CPM facilitation, this did not withstand the Bonferroni correction for multiple comparisons (*p* = 0.044).

As shown in Figure 2C, there was a medium effect size correlation between the CPM response and the pressure of the CS, but this failed to reach significance with our sample size (*rho* = 0.207, *p* = 0.059). Similarly, there was no correlation between the CPM response and both the pain rating of the pressure cuff (*rho = *−0.065, *p* = 0.56, Figure 3D) and the pain rating at baseline (*rho = *0.071, *p* = 0.53, Figure 3E).

#### 3.4.5 Time of CPM visit

As shown in Figure 2F, there was variation in the time the CPM visit began. However, there was no significant correlation between the CPM response and the time of CPM visit (*rho = *0.166, *p* = 0.13).

#### 3.4.6 Medications used

Only six (23.1%, Supplementary Table S1A) control participants and seven (11.9%, Supplementary Table 1B) CPP participants were not taking any medications at the time of the CPM visit. There was no significant difference in the proportion not taking medications (*p* = 0.204). The most common medications used were multivitamins/supplements (34.6%), anti-depressants/mood stabilizers (30.8%) and antihistamines (19.2%) (Supplementary Table 3C).

### 3.5 Quantitative sensory testing

In healthy control participants, no significant sensory perturbations in any of the QST measures were found, relative to the healthy reference data, in any of the CPM response groups, as shown in Figure 3A. There was a significant difference between the facilitatory and non-responder as well as between facilitatory and inhibitory for warm detection threshold (WDT) but these did not withstand multiple- comparisons correction (*p* = 0.044 and *p* = 0.020 respectively, Figure 3C). There were no significant differences between the CPM response groups in any of the QST measures.

In CPP participants, there was a significant loss of function in the vibration detection threshold within the facilitatory response group at the group level compared with the healthy reference data, Figure 3B. However, there were no significant differences between the CPM response groups in any of the QST measures.

Furthermore, there was no significant difference between inhibitors and facilitators when looking at mean Z scores for nociceptive or mean Z scores for non-nociceptive QST parameters in both CPP participants and CON.

## 4 Discussion

This study aimed to compare CPM between female pain free controls and those with chronic pelvic pain. Contrary to similar studies in the literature, e.g., on fibromyalgia or chronic widespread pain (33) we found no significant differences in the CPM effect between groups. Importantly even in our pain free controls we could only demonstrate a “true CPM effect” in 23% of participants. Despite having a well-phenotyped cohort, we were unable to find a strong association between CPM and any single phenotypic factor which had previously been suggested to impact the CPM response (14, 34–36).

### 4.1 Choice of conditioned pain modulation experimental paradigm

While there is no gold-standard CPM paradigm, we followed recommendations for best practice (13, 31, 37, 38). Importantly, the second stimulus was delivered after the CS (sequentially) rather than in parallel. While this reduces the observed effect sizes it serves to minimize the confounding effects of distraction (12). We ensured standardisation across study sites: coordinated researcher training, adherence to a pre-prepared script and use of the same equipment (29). We are therefore confident that our results are not due to deviations in the study protocol.

We used a pressure cuff paradigm as CS, an approach which has been suggested as more likely to produce CPM inhibition in healthy controls than a heat-based approach (10). However, we were only able to elicit an inhibitory CPM response in 15.4% of our control cohort. Whilst some research has suggested the pressure cuff is a more reliable CS than heat or iced water (39) and that a PPT as TS has excellent reliability (14, 40), the cold pressor test has been shown to elicit excellent ICC (41). Nonetheless, we were only able to demonstrate fair reliability of our paradigm (42) using the PPT, however this result may have differed had another paradigm been employed (38). Future studies using other variants of validated CPM paradigms might arrive at different conclusions.

Altered sensory function at the test site has been shown to be associated with enhanced inhibition in neuropathic pain (43–45) and thus the choice of stimulus location in chronic pain cohorts is likely to be important. We have previously demonstrated altered sensory profiles on the abdomen in the majority of our CPP participants (32) however our PPT was delivered to the foot and we found no significant differences in nociceptive QST measures on the foot between participants exhibiting CPM inhibition or facilitation. Interestingly, however, there was some evidence of altered sensory function on the foot in facilitatory responders (both CPP and control participants) in exploratory analyses (Figure 3).

It is interesting to note that contrary to expectations the ischemic pain threshold was higher in the CPP group than the control group. However, the pain intensity rating at the end of the 60 s stimulus duration was also higher in the CPP group, meaning that ischaemic pain increased at a higher rate than in controls once the pain threshold was reached.

### 4.2 Lack of a “true CPM effect” in the majority of the healthy controls

We employed the standard error of measurment +/−2SEm threshold as a robust statistical measure of a “true” CPM effect (14, 15). Previous studies using this methodology with similar aged participants demonstrated a true inhibitory effect in 44%–59% of controls (14), with a higher proportion in paediatric participants (75%) (45). Although the intensity of the CS has been reported as influencing the CPM response (46) the standardised effect sizes of the influence of either cuff pressure or reported pain intensity of the CS were too small to reach significance with our sample size.

Unsurprisingly, some of our controls (*N* = 9) described background pain (e.g., headache) on the day of testing, however, this was mild (mean 1.4/10, SD 0.7). Importantly, the intensity of background pain on the day of testing was not related to CPM effect in either the controls or the CPP participants.

### 4.3 CPM and participant characteristics

Given the (frequently conflicting) literature on the variety of participant characteristics that can influence the CPM response (45), we explored these relationships within our data. We found no correlations between the CPM response and widespread pain characterization. Additionally, we found no correlation between the CPM response and neither state anxiety [aligning with other studies (45)] nor pain catastrophising (contrary to other literature (47–49). The unpleasantness of the CS has also been described as contributing to CPM effect (31, 50). We did not specifically measure this, however, researchers across all three sites noted that participants found the CPM paradigm particularly unpleasant, especially amongst a battery of other, more well-tolerated psychophysical testing paradigms (i.e., DFNS QST protocol, non-invasive bladder sensitivity testing and ANS testing). Thus, CS unpleasantness may have contributed to increased CPM inefficiency and the low proportion of our cohort exhibiting CPM inhibition.

We did not assess hormone levels on the day of the CPM paradigm, nor did we exclude participants who were currently on a form of hormonal contraceptive as this is a mainstay of the treatment of CPP. Therefore, our data adds little to the understanding of the influence of hormones on CPM (4, 51, 52). However, we did not see a clear suggestion from our data that cycle phase or exogenous hormone use influenced CPM effect.

Given that centrally-acting medications, particularly those acting on serotonin pathways, may impact on CPM (53–56), we explored the medication use in our cohort. Again, our sample size prohibits detailed analysis, however there was no clear relationship seen.

### 4.5 Limitations

Our study was carefully designed, using one of the recommended CPM paradigms, however, there remain limitations which must be considered when drawing conclusions from these results. Whilst we did identify a fair reliability between PPT3average—PPT1average and PPT2average -PPT1average this may have been influenced by the short time (10 mins) between the sessions.

Moreover, performing the study in three different countries, while the Control group was only recruited from Boston may have impacted the results, as there are evidence that ethnic differences play a role in CPM response (57, 58). Additionally, variations in BMI (higher in the CPP group but not significant) or age, could also have further contributed to the variability in our findings (59, 60).

Additionally, there is heterogeneity in the definition of ‘healthy controls’ in CPM studies (61). Many studies exclude participants based on comorbidities, medication use and other physical characteristics. It has been proposed that such heterogeneity contributes to variability in CPM results (35, 62, 63). We only excluded participants with moderate-severe CPP (>=3/10), and those with endometriosis, dysmenorrhoea or urinary symptoms. Analysis of our baseline questionnaire data has shown the presence of comorbidities within the control group (albeit with a lower frequency than in the CPP groups) (21), however, very few participants had painful conditions which would alter somatosensory functioning, as shown in Supplementary Table 2. We therefore consider it unlikely that the presence of other diagnoses contributed to so few participants with intact CPM in our control group. This lack of effect in the positive control group remains a limitation. Other validated CPM paradigms may yield different findings in the future.

We acknowledge that the sample for the study is smaller than it would be required to prove no difference between patients and controls or a more detailed analysis into the factors affecting the lack of difference. The present study is based on Phase III of the larger TRiPP project, in which participant recruitment has been significantly affected by the COVID-19 restrictions leading to recruiting fewer participants than intended. However, only 23.1% of the control group exhibited a “true CPM effect”, indicating that this information remains valuable despite the limitations.

Defining the CPP group using different underlying pathologies could be considered a limitation. However, the aim of the TRiPP project is to reconceptualise the conditions (EAP and BPS) in the context of multisystem dysfunction similar to other chronic pain conditions to identify more meaningful subgroups and move away from the end-organ approach. Currently we do not have any biological explanation as to why CPM would differ between these conditions but future studies could consider revisiting this in the future if new evidence become available.

## 5 Conclusions

Contrary to our hypothesis, we were unable to demonstrate a significant difference in the CPM effect between control women and those with CPP. Importantly, only a small proportion of our controls exhibited CPM inhibition. Interestingly, we were unable to identify phenotypic features relating to the presence or magnitude of a CPM effect. Although CPM in chronic pain populations is of major theoretical mechanistic interest because of its prominence in the nociplastic pain concept (63), the lack of an established assessment standard leads us to question its added value in current clinical research.

## Data Availability

The raw data supporting the conclusions of this article will be made available by the authors, without undue reservation.
