# Supplementary material for: Exploring the value of a well-established conditioned pain modulation paradigm in women: a Translational Research in Pelvic Pain (TRiPP) study
Source: Front Pain Res (Lausanne). 2025 Mar 12;6:1439563. doi: 10.3389/fpain.2025.1439563 (PMC11936887; doi:10.3389/fpain.2025.1439563)
Supplement: Supplementary file 1 [file Datasheet1.docx]

**Supplementary**

**Supplementary Figure 1.** TRiPP study design flow chart, taken with permission from the TRiPP baseline characteristics paper. Phase I; data was collected prior to TRIPP commencing (in Oxford and Boston) or at the time of TRiPP study commencement (in Porto). Phase II; Additional questionnaires (either online or on paper) at each study site were administered. Phase III/IV; Carried out by trained researchers at the respective site(s). EAP; endometriosis-associated pain, EABP; endometriosis-associated pain and bladder pain symptoms, BPS; bladder pain syndrome, PP; pelvic pain, Con; control, EndOx; study in Oxford to identify biomarkers for endometriosis, BCE; Boston Centre for Endometriosis, IBMC; Instituto de Biologia Molecular e Celular, fMRI; functional magnetic resonance imaging. fMRI was solely performed in Oxford.


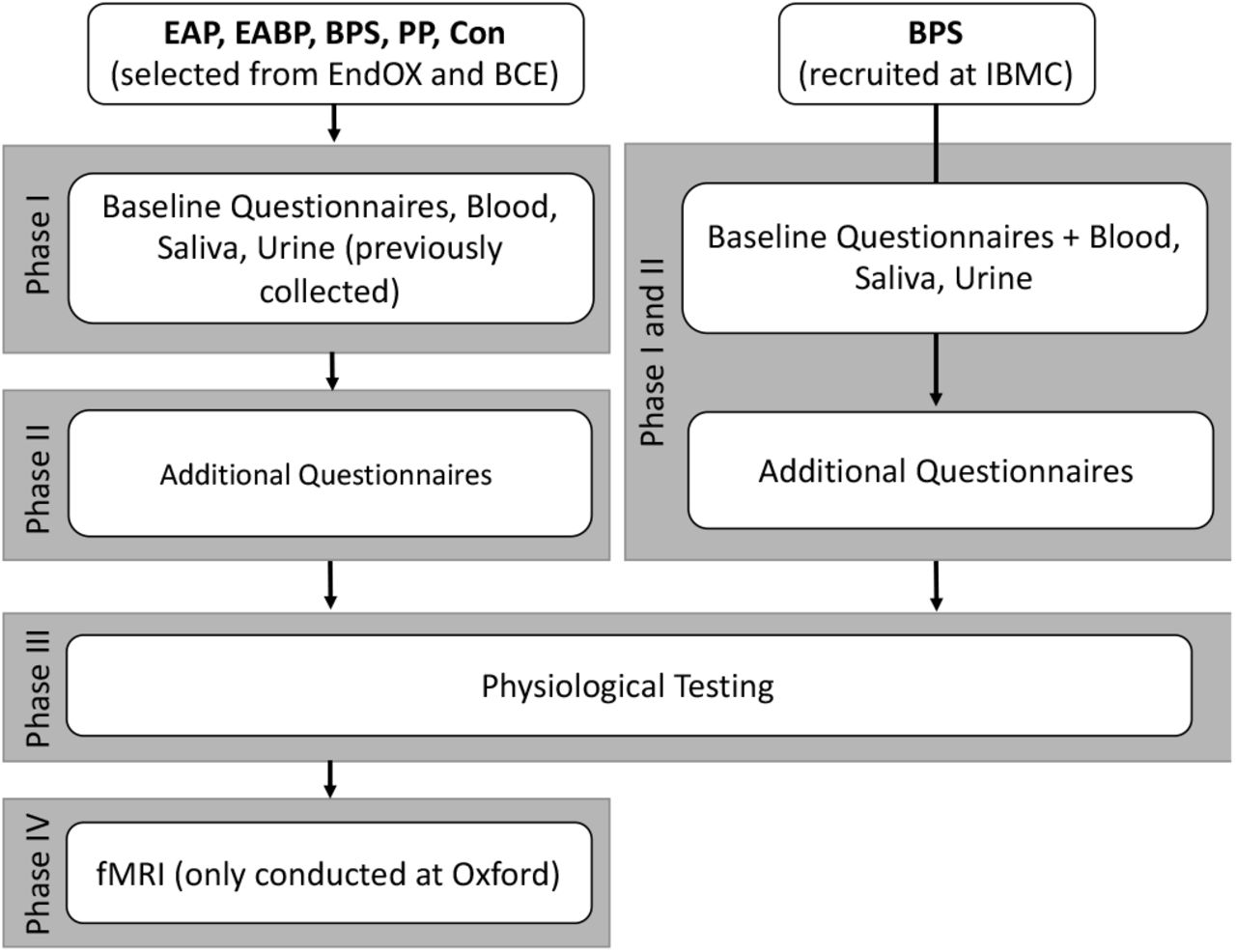


**Supplementary Figure 2.** CPM paradigm sequence of PPT (pressure pain threshold) and CS (conditioning stimulus).

**
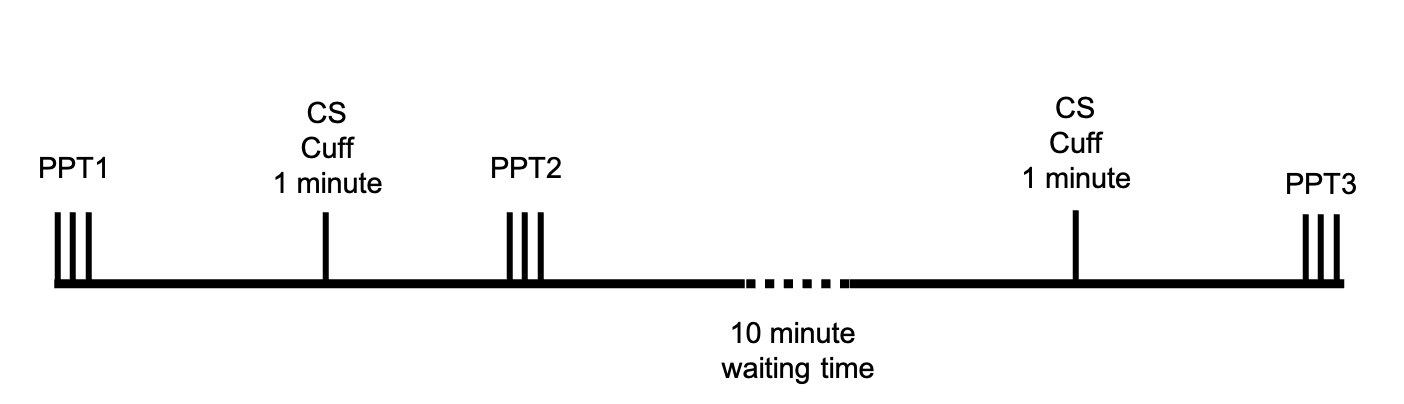
**

**Table 1.** Number (%) of participants using at least one type of medication, by medication class. Medication names have been outlined below. *It is possible for participants to have been taking more than one of the medications listed in each medication class, but they were counted only once in the number (%) using medications within that class. SNRI: serotonin and norepinephrine reuptake inhibitor; SSRI: selective serotonin reuptake inhibitor

1. Controls only (n=26)

| **Medication Class** | **Medication Name** | **N (%) participants using at least one type of medication from the medication class*** |
| --- | --- | --- |
| Anti-depressants/Mood stabilisers | Duloxetine (SNRI), Bupropion, Escitalopram (SSRI), Venlafaxine (SNRI), Citalopram (SSRI), Lithium, Nonbenzodiazepines | 8/26 (30.8) |
| ADHD Medication | Adderall | 1/26 (3.8) |
| Analgesics | Ibuprofen | 1/26 (3.8) |
| Anti-convulsant | Lamotrigine | 1/26 (3.8) |
| Antihistamine | Loratadine, Hydroxyine, Diphenhydramine, Cetirizine, Fluticasone Propionate Nasal Spray | 5/26 (19.2) |
| Beta Blockers | Propranolol | 1/26 (3.8) |
| Thyroid Medication | Levothyroxine | 1/26 (3.8) |

^a^6/26 (23.1%) of control participants were not taking any medications

1. CPP only (n=59)

| **Medication Class** | **Medication Name** | **N (%) participants using at least one type of medication from the medication class*** |
| --- | --- | --- |
| Anti-depressants/Mood stabilisers | Duloxetine (SNRI), Lamotrigine, Fluvoxamine (SSRI), Citalopram (SSRI), Sertaline (SSRI), Buspar, Abilify Maintena, Fluoxetine (SSRI), Trazodone AC, Victan, Diazepam, Sedoxil, Lexotan, Bialzepam, Ansilor, Mirtazapine, | 17/59 (28.9) |
| ADHD Medication | Strattera, Metadate | 2/59 (3.4) |
| Analgesic or anti-inflammatory medication | Ibuprofen, Orilicssa, Meloxicam, Nortryptiline, Humira pen, Budesonide, Benuron, Pregabalin, Paracetamol, Codeine, Amitriptyline, Oramorph, Sulfasalazine, | 15/59 (25.4) |
| Anti-histamine | Zyrtec, Flonase, Fexofenadine, Nytol, Generic Hayfever Relief Medication, Cetirizine, Inhaler | 10/59 (16.9) |
| Leukotriene receptor antagonist | Singulair (for asthma) | 1/59 (1.7) |
| Beta Blockers | Propranolol, Nebilet | 2/59 (3.4) |
| Antifungals or Antibiotics | Ciprofloxacin | 1/59 (1.7) |
| Thyroid Medication | Levothyroxine, Thyroxine | 3/59 (5.8) |
| Aromatase Inhibitors | Letrozole | 1/59 (1.7) |
| Sleeping Medication | Zolpidem | 2/59 (3.4) |
| Weight-loss medication | Saxenda injection | 1/59 (1.7) |
| Anti-epileptic medication | Topiramate | 1/59 (1.7) |
| Migraine Medication | Excedrin, Ajovy, Sumatriptan Succinate | 2/59 (3.4) |
| Opioid Use Disorder Management | Naltrexone | 1/59 (1.7) |

^a^7/59 (11.9%) participants were not currently taking any medication

1. CPP participants by CPM group. N of Participants in each CPM group using centrally acting medications.

| **Medication Class** | **Inhibitory group (N (%))** | **Non-response group (N (%))** | **Facilitatory group (N (%))** |
| --- | --- | --- | --- |
| Anti-depressants/Mood stabilisers | 3 (27.3) | 13 (32.5) | 1 (12.5) |
| Beta Blockers | 0 | 2 (5) | 0 |
| ADHD Medication | 1 (9.1) | 0 | 0 |
| **Total centrally acting medications** | **4 (36.4)** | **15 (37.5)** | **1 (12.5)** |

**Supplementary Table 2.** Comorbid conditions by CPM response group in healthy controls. Participants were asked to select from a list of predefined conditions which condition they had ever received a diagnosis of.

|  | **Facilitatory (/2)** | **Non-responder (/20)** | **Inhibitory (/4)** |
| --- | --- | --- | --- |
| Anxiety requiring medication or therapy | 1 | 5 | 3 |
| Asthma | 0 | 2 | 1 |
| Cardiovascular disease | 0 | 0 | 1 |
| Crohn’s disease | 0 | 0 | 0 |
| Chronic fatigue syndrome | 0 | 0 | 0 |
| Deafness/difficulty hearing | 0 | 0 | 1 |
| Depression requiring medication or therapy | 0 | 4 | 1 |
| Diabetes requiring diet control | 0 | 0 | 0 |
| Diabetes requiring insulin or tablets | 0 | 0 | 0 |
| Eczema | 0 | 0 | 2 |
| Uterine fibroids | 1 | 0 | 0 |
| Glandular fever | 0 | 0 | 0 |
| Graves’ disease | 0 | 0 | 0 |
| Hashimoto’s disease | 0 | 0 | 0 |
| High blood pressure | 0 | 0 | 0 |
| Irritable bowel syndrome | 0 | 0 | 0 |
| Migraine | 0 | 1 | 0 |
| Mitral valve prolapse | 0 | 3 | 0 |
| Multiple sclerosis | 0 | 0 | 0 |
| Painful bladder syndrome | 0 | 0 | 0 |
| Pelvic inflammatory disease | 0 | 0 | 0 |
| Polycystic ovarian syndrome | 0 | 0 | 0 |
| Rheumatoid arthritis | 0 | 0 | 0 |
| Scoliosis | 0 | 0 | 1 |
| Other spine problems | 0 | 0 | 0 |
| Sjogren’s syndrome | 0 | 0 | 0 |
| Systemic lupus erythematosus | 0 | 0 | 0 |
| Thyroid disease | 0 | 1 | 0 |
| Ulcerative colitis | 0 | 0 | 0 |
| Number (%) with at least one condition | 2/2 (100%) | 11/20 (55%) | 4/4 (100%) |
